# SCPEP1^+^ basal cells are associated with the remodeling of oxidative stress signaling networks in idiopathic pulmonary fibrosis

**DOI:** 10.3389/fimmu.2025.1676086

**Published:** 2025-12-08

**Authors:** Xiang Zhou, Tong Lu, Ran Xu, Chenghao Wang, Simiao Chen, Jing Chen, Xiaoyan Chang, Meifeng Li, Jiaxin Shi, Chengyu Xu, Yupeng Zhao, Bo Peng, Jiaying Zhao, Linyou Zhang

**Affiliations:** 1 Department of Thoracic Surgery, The Second Affiliated Hospital of Harbin Medical University, Harbin, Hei Longjiang, China; 2 Department of Thoracic Surgery, Ruijin Hospital, Shanghai Jiao Tong University School of Medicine, Shanghai, China; 3 Department of Nephrology, The Second Affiliated Hospital of Harbin Medical University, Harbin, Hei Longjiang, China

**Keywords:** idiopathic pulmonary fibrosis, oxidative stress, single-cell RNA sequencing, spatialtranscriptomics, machine learning, SCPEP1

## Abstract

**Background:**

Idiopathic pulmonary fibrosis (IPF) is a chronic and fatal interstitial lung disease marked by progressive extracellular matrix accumulation and irreversible lung architecture remodeling. Oxidative stress (OS) plays a crucial role in IPF pathogenesis, yet its role across distinct cellular compartments and tissue microenvironments remains incompletely characterized.

**Methods:**

We integrated single-cell RNA sequencing (scRNA-seq), spatial transcriptomics (stRNA-seq), and bulk RNA-seq datasets to comprehensively characterize oxidative stress activity across cellular and tissue scales in IPF lungs. Oxidative stress scores were calculated using multiple enrichment algorithms, and machine learning models (LASSO, Random Forest, Boruta, Bayesian, LVQ, Treebag) were applied to identify robust OS-related diagnostic biomarkers. Expression patterns were validated in public datasets and a bleomycin-induced C57BL/6 mouse model. Cell-cell communication and gene regulatory pathways were further explored using CellChat and pseudotime trajectory analysis.

**Results:**

Oxidative stress activity was significantly elevated in IPF lung tissue and specifically enriched in basal cells. Among 71 candidate OS-related genes, SCPEP1 emerged as the most robust biomarker, consistently upregulated across multiple datasets and experimental validation, with an AUC of 0.857 in the training cohort. SCPEP1 expression was spatially confined to airway-adjacent regions and highly specific to basal cells. SCPEP1^+^ basal cells exhibited transcriptional reprogramming enriched in Wnt signaling and developmental pathways, dynamic expression during early pseudotime progression, and engaged in multifaceted interactions with immune and stromal cells through pro-fibrotic and inflammatory signaling axes such as MIF-CD74, MDK-NCL, and ICAM1-ITGAL. Translationally, these findings may help prioritize redox-sensitive pathways and ligand–receptor interactions for further investigation. While SCPEP1 appears to be a promising candidate, its potential for patient stratification or therapeutic intervention remains to be confirmed through functional studies.

**Conclusion:**

Our multi-omics integration revealed SCPEP1^+^ basal cells as central oxidative stress responders and communication hubs in IPF. These findings provide insights into ROS-driven epithelial remodeling and highlight SCPEP1 as a potential contributor to disease-associated pathways that warrants further exploration for its diagnostic or therapeutic relevance.

## Introduction

Pulmonary fibrosis (PF) is a chronic, progressive interstitial lung disease characterized by excessive deposition of extracellular matrix (ECM) and collagen in the pulmonary stroma. These pathological changes lead to alveolar destruction, impaired gas exchange, and ultimately respiratory failure (1, 2). In recent years, PF has emerged as a significant global public health concern, with rising incidence and mortality rates that severely compromise patients’ quality of life and overall prognosis (3–6). Although advances have been made in understanding the pathogenesis of PF, and antifibrotic agents such as pirfenidone and nintedanib have been introduced into clinical practice, the median survival after diagnosis remains poor, typically ranging from 3 to 5 years (7–9). Late-phase trials are mixed: pamrevlumab and PRM-151 failed in phase 3, whereas nerandomilast reduced FVC decline and inhaled treprostinil met its primary endpoint-signaling progress yet persistent unmet need (10, 11).Therefore, elucidating the cellular and molecular mechanisms underlying PF and identifying novel therapeutic targets and reliable diagnostic or prognostic biomarkers are urgently needed.

Oxidative stress (OS) plays a pivotal role in the pathogenesis of PF, particularly idiopathic pulmonary fibrosis (IPF). It reflects an imbalance between the generation of reactive oxygen species (ROS) and the antioxidant defense system in lung tissues (12, 13). Under physiological conditions, ROS are maintained at controlled levels and participate in normal cellular signaling and host defense processes (14, 15). However, during pathological conditions such as PF, persistent lung injury, chronic inflammation, and mitochondrial dysfunction lead to excessive ROS production, overwhelming the antioxidant capacity. This results in damage to alveolar epithelial and endothelial cells, and promotes epithelial-to-mesenchymal transition (EMT) and fibroblast-to-myofibroblast transition (FMT), thereby accelerating ECM deposition and tissue remodeling and contributing to disease progression (16–18).

Although targeting oxidative stress has been proposed as a therapeutic strategy for PF, current clinical outcomes remain unsatisfactory (19). Notably, there is a lack of comprehensive studies on the cell-type-specific oxidative stress landscape in PF and limited characterization of OS-related genes and pathways. A systematic investigation of OS-associated gene expression profiles and functional roles, with an emphasis on cellular specificity, may provide new molecular targets for PF therapy and inform the development of innovative therapeutic strategies.

With the rapid advancement of high-throughput sequencing technologies, single-cell RNA sequencing (scRNA-seq) has become a powerful tool in biomedical research. It enables high-resolution characterization of cellular heterogeneity and has significantly contributed to the elucidation of disease pathogenesis across multiple contexts (20, 21). Meanwhile, the emergence of spatial transcriptomics (ST) allows researchers to investigate gene expression patterns *in situ* with spatial context, further improving our understanding of regional pathology and local microenvironments (22, 23). In parallel, the integration of bioinformatics with machine learning has enabled more accurate identification of disease-related biomarkers and therapeutic targets (24).

**Figure 1:**
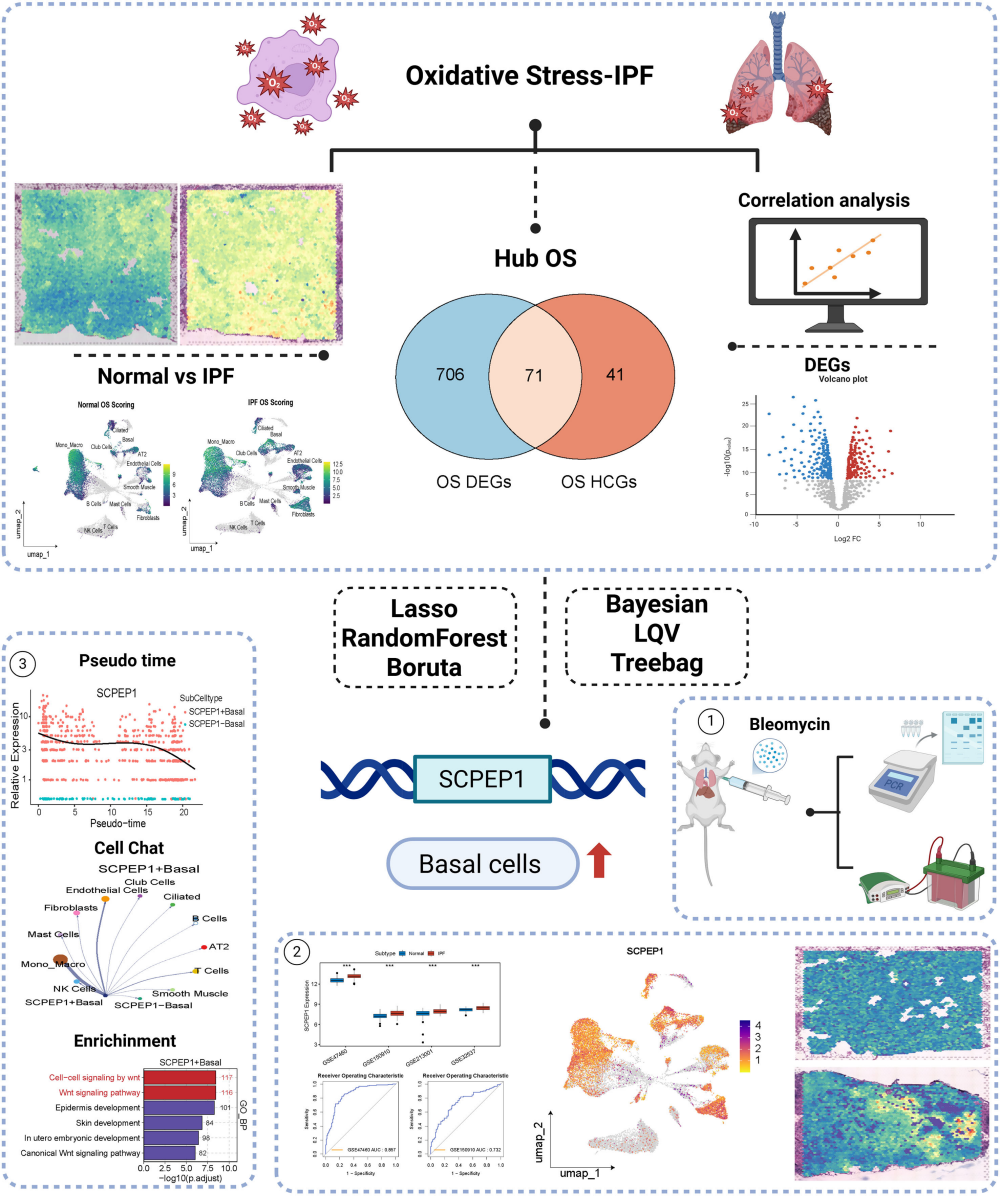
Graphical abstract of study work flow.

Although oxidative stress has been implicated in IPF by numerous studies, its precise functional role at the single-cell and spatial levels remains incompletely understood. In this study, we comprehensively integrated scRNA-seq, bulk RNA-seq, and spatial transcriptomics to characterize oxidative stress dynamics in IPF across cellular, tissue-wide, and spatial dimensions. Using multiple machine learning algorithms, we prioritized key oxidative stress-related genes and pathways. Graphical abstract (**Figure 1**) shows our all design. Furthermore, we validated our findings *in vivo* using a bleomycin-induced C57BL/6 mouse model. Our study not only unveils novel oxidative stress mechanisms in IPF, but also provides robust theoretical support for the development of precise diagnostic and therapeutic strategies for fibrotic lung diseases.

## Materials and methods

### Data acquisition

To investigate oxidative stress (OS)–related alterations during disease progression in IPF patients, we retrieved spatial transcriptomics data (S-BSST1410) (25) from the BioStudies database (https://www.ebi.ac.uk/biostudies/), which included spatial transcriptomics sequencing samples from 6 patients with severe IPF and 6 healthy controls. We also downloaded IPF-related public datasets from the Gene Expression Omnibus (GEO, https://www.ncbi.nlm.nih.gov/geo/), including:GSE128033 (26, 27)(scRNA-seq with 8 IPF patients and 10 healthy individuals)GSE47460 (28) (bulk RNA-seq with 168 IPF and 108 healthy lung samples), GSE32537 (29) (bulk RNA-seq data with 119 IPF and 50 healthy lung samples), GSE150910 (30) (bulk RNA-seq data with 103 IPF and 103 healthy lung samples), GSE213001 (31)(bulk RNA-seq data with 62 IPF and 42 healthy lung samples). Finally, a total of 807 oxidative stress-related genes were extracted from a previously published study and are listed in **Supplementary Table S1** (32).

### Preprocessing of stRNA-seq and scRNA-seq data

For spatial transcriptomic (stRNA-seq) data, individual samples were processed using the Seurat R package(v4.3.0) to construct Seurat objects. These objects were then merged using the merge function. The samples were grouped by their original identifiers (orig.ident), and spatial distributions of features across spots were visualized using the SpatialFeaturePlot function. To estimate the cellular composition within each spot, we performed deconvolution analysis using the SPOTlight package.

For single-cell RNA sequencing (scRNA-seq) data from IPF patients, preprocessing and analysis were performed using the Seurat R package as well. The NormalizeData function was applied with the “LogNormalize” method to normalize raw gene expression data, which were then converted into Seurat objects. Quality control (QC) was ensured by calculating the percentages of ribosomal and erythrocyte-related genes, followed by filtering out low-quality cells. Specifically, we excluded cells with fewer than 100 or more than 8000 detected genes, ribosomal gene content exceeding 10%, or erythrocyte gene content above 3%. Next, we identified the top 3000 most variable genes using the FindVariableFeatures function to serve as the basis for normalization and downstream analysis. Principal component analysis (PCA) was performed via ScaleData and RunPCA functions. To remove batch effects, we applied the Harmony algorithm for normalization. Dimensionality reduction was carried out using the UMAP algorithm, followed by clustering with the FindNeighbors and FindClusters functions (resolution = 1.0). Cell type annotation was performed manually based on canonical marker genes. Visualization of major cell types and subtypes was conducted using Idents, DimPlot, the scCustomize package, and the SCP package.

### Oxidative stress activity characterization across cell types

To evaluate oxidative stress (OS) activity, we employed the irGSEA R package to calculate OS scores based on the expression of 807 OS-related genes. Four enrichment scoring methods-AUCell, UCell, singscore, and AddModuleScore were applied to both spatial transcriptomic and single-cell RNA-seq datasets. Each score was standardized using the scale function, and the sum of the four normalized scores was used as a composite index, referred to as the Scoring, to quantify OS activity.

In spatial transcriptomic (stRNA-seq) data, OS scores were calculated for each spatial spot and subsequently summed to generate a composite OS score (OSscore). Spatial visualization of OSscore was performed using the SpatialFeaturePlot function in Seurat to compare oxidative stress levels across different tissue regions. Differences in OS scores between IPF and normal tissues were assessed using the FeatureStatPlot function from the SCP package.

In single-cell (scRNA-seq) data, OS scoring distributions were calculated and visualized across different cell types. Based on the median Scoring value of all cells, cells were stratified into high OS activity and low OS activity groups. Differential expression analysis between these two groups was conducted using the RunDEtest function from the SCP package. OS-related genes with P < 0.05 and |logFC| > 1 were designated as OS-related differentially expressed genes (OS DEGs).In addition, we performed correlation analysis between each expressed gene and Scoring, selecting genes with P < 0.05 and correlation coefficient > 0.5. The intersection of these correlated genes with OS DEGs was used for univariate logistic regression analysis. Genes with significant predictive power were identified as high-risk OS genes and designated as candidate hub OS biomarkers for IPF diagnosis.

### Muti-machine learning driven discovery of hub OS related genes in IPF

To further identify high-value oxidative stress (OS)-related biomarkers in IPF, we used GSE47460 as the training dataset and GSE150910, GSE213001, and GSE32537 as independent validation cohorts with z-scored methods to avoid leakage. A total of six machine learning algorithms were employed to screen for hub OS-related genes, including Lasso, Random Forest, Boruta, Bayesian modeling, Learning Vector Quantization (LQV), and Treebag. All models were evaluated using appropriate cross-validation strategies to ensure robustness and generalizability.

LASSO introduces an L1 penalty to shrink some coefficients to zero, thereby enabling effective feature selection and dimensionality reduction. The penalty parameter λ was optimized using 10-fold cross-validation via the cv.glmnet function, and genes with non-zero coefficients were retained. Random Forest constructs an ensemble of decision trees using random data subsets and aggregates their outputs. We grew 500 trees with default mtry and calculated variable importance. To stabilize feature selection, we applied 30-fold cross-validation and retained genes with median importance values above zero across folds. Boruta, built upon the RF framework using the Boruta package, iteratively compared each feature’s importance with that of randomized “shadow features.” Although Boruta does not incorporate formal cross-validation, its randomized iterative scheme was repeated 100 times to ensure stability. Features with statistically higher Z-scores than shadow max were confirmed. Bayesian was executed using the arm package. Given its strength in low-sample, high-dimensional settings, we trained models using a 30-fold cross-validation scheme and incorporated weakly informative priors. Predictive features were selected based on posterior means and inclusion probabilities. Learning Vector Quantization (LVQ) was applied via the caret package, using the lvq method. Feature selection was based on relative importance across models trained with 5-fold cross-validation repeated 3 times. Top-ranked genes were retained based on averaged scaled importance. Lastly, Treebag (bagged trees) was implemented using the treebag method from caret. We trained ensembles of decision trees on bootstrapped samples and evaluated feature importance using 30-fold cross-validation. Genes consistently contributing to classification accuracy were retained.

We subsequently selected Final OS genes as candidate diagnostic biomarkers for IPF by identifying hub OS genes that were consistently selected by all 6 algorithms. The predictive performance of the Final OS genes was assessed by receiver operating characteristic (ROC) curve analysis in both the training and validation cohorts. We also examined their differential expression levels between IPF patients and healthy controls to confirm their diagnostic relevance.

### Construction of bleomycin-induced IPF model in C57BL/6 Mice

Eight-week-old female C57BL/6NCrl mice (purchased from the Animal Experimental Center of the Second Affiliated Hospital of Harbin Medical University) were used in this study. Mice were anesthetized via intraperitoneal injection of avertin (2, 2, 2-tribromoethanol, 250 mg/kg). A small midline incision was made at the neck to expose the trachea. Each mouse received an intratracheal instillation of 30 μL bleomycin (BLM, Selleck, S1214, 3 mg/kg) dissolved in sterile saline. Control mice received an equivalent volume of saline. Mice were housed in standard Macrolon type III cages with aspen wood chip bedding (Rettenmeier and Sohne) under a 12 h light/12 h dark cycle at 21 ± 1°C and 55 ± 15% relative humidity, with ad libitum access to food and tap water.

On day 28 after treatment, lung tissues were collected (IPF group: n = 5; control group: n = 5). During tissue collection, mice were re-anesthetized with intraperitoneal injection of Avertin (2, 2, 2-tribromoethanol, 250 mg/kg) and subsequently euthanized by cervical dislocation in accordance with institutional ethical guidelines. A longitudinal incision was made from the abdomen to the mandible to expose the thoracic cavity. The left atrial appendage was opened, and physiological saline at room temperature was gently perfused through the heart to flush the systemic and pulmonary circulation. The lungs were then carefully harvested, trimmed, placed into cryogenic vials, snap-frozen in liquid nitrogen, and stored at −80 °C for subsequent molecular analyses. For paraffin embedding, lung tissues were fixed by tracheal instillation of 4% paraformaldehyde immediately after saline perfusion, followed by immersion in 4% paraformaldehyde for further fixation prior to histological sectioning and staining.

### Histological staining

Paraffin-embedded tissue sections were prepared by Servicebio (Wuhan, China) following standard operating procedures, including tissue fixation, dehydration, embedding, and sectioning. Then, hematoxylin and eosin (H&E) staining was performed using a commercial HD staining kit (Servicebio, G1076). After deparaffinization and rehydration through graded ethanol solutions, sections were pretreated and stained with hematoxylin for 3–5 minutes, followed by differentiation, bluing, and eosin staining for 15 seconds. Slides were then dehydrated in absolute ethanol and butanol, cleared in xylene, and mounted with neutral balsam. Nuclei were stained blue, while cytoplasm appeared red.

Masson’s trichrome staining was conducted using a staining kit (Servicebio, G1006). Following dewaxing and hydration, sections were incubated overnight in Masson A solution, then stained sequentially with B+C mixture (1:1), differentiated, and further stained with Masson D, E, and F reagents. After rinsing in glacial acetic acid and dehydration, slides were cleared in xylene and sealed. Collagen fibers appeared blue, whereas muscle fibers, fibrin, and red blood cells stained red.

All stained slides were examined under a Nikon ECLIPSE E100 microscope, and images were captured using the Nikon DS-U3 imaging system for further analysis.

### Western blot

Lung tissues from IPF model mice and control mice were homogenized via ultrasonic disruption. Total protein was extracted using RIPA lysis buffer (Beyotime Biotechnology, Shanghai, China) supplemented with a protease inhibitor cocktail and PMSF (Beyotime Biotechnology, Shanghai, China). Protein concentrations were determined using a BCA Protein Assay Kit (Thermo Fisher Scientific, CA, USA).For western blot analysis, samples were mixed with appropriate volumes of SDS-PAGE loading buffer, denatured by boiling, and subjected to electrophoresis. Proteins were separated on 10% SDS-PAGE gels, with a pre-stained protein ladder (Thermo Fisher Scientific, CA, USA) used as the molecular weight marker. After electrophoresis, proteins were transferred to PVDF membranes (Millipore, USA) and blocked with rapid blocking buffer (PS108, Vazyme) for 15 minutes. Membranes were incubated with primary antibodies overnight at 4°C, followed by incubation with HRP-conjugated secondary antibodies at room temperature for 1 hours. Protein bands were visualized using an enhanced chemiluminescence (ECL) kit (MA0186, Meilunbio, Liaoning, China).

The primary antibodies used included anti-SCPEP1 antibody (1:3000, 29781-1-AP, Proteintech, Wuhan, China) and anti-β-actin antibody (1:20000, Proteintech, Wuhan, China). The secondary antibody was HRP-conjugated goat anti-rabbit IgG (1:10000, Proteintech, Wuhan, China). All band intensities were quantified using ImageJ software (Rawak Software Inc., Stuttgart, Germany).

### Real-time quantitative PCR

Total RNA was extracted from lung tissues using FreeZol Reagent (R711-01, Vazyme, Nanjing, China) according to the manufacturer’s instructions. The concentration and purity of the extracted RNA were assessed using a NanoDrop spectrophotometer, with acceptable A260/A280 ratios between 1.8 and 2.0. RNA integrity was verified by agarose gel electrophoresis.For removal of genomic DNA and reverse transcription, we used the HiScript^®^ III RT SuperMix for qPCR (+gDNA wiper) kit (R323-01, Vazyme, Nanjing, China), following the recommended protocol provided by the manufacturer.

Quantitative PCR was performed using ChamQ Universal SYBR qPCR Master Mix (Q711-02, Vazyme, Nanjing, China) on a Roche LightCycler 480 system. Each 20 μL PCR reaction contained 10 μL SYBR mix, 0.4 μL forward primer (10 μM), 0.4 μL reverse primer (10 μM), 0.4 μL diluted cDNA, and 8.8 μL nuclease-free water. The amplification protocol consisted of an initial denaturation at 95°C for 30 seconds, followed by 40 cycles of denaturation at 95°C for 5 seconds and annealing/extension at 60°C for 30 seconds.

Relative expression levels of target genes were calculated using the 2^^−ΔΔCt^ method, with GAPDH serving as the internal control. All reactions were performed in technical triplicates and repeated in at least two independent experiments to ensure data reliability and reproducibility. The primer sequences used for all target genes are listed in **Supplementary Table S3**.

### Mapping final OS expression across major cell populations

Subsequently, we utilized the FeaturePlot_scCustom function from the scCustomize R package to generate feature plots showing the distribution of Final OS expression across cell types. In parallel, we used the FeatureStatPlot function from the SCP package to statistically compare expression levels among different cell populations.In the top-ranked cell type with the highest expression level of Final OS, we further distinguished Final OS positive and Final OS negative subpopulations based on gene expression status.

### Pseudotime analysis

To explore the dynamic changes in Final OS gene expression during cell differentiation or state transitions, we performed pseudotime analysis using the Monocle R package. First, we extracted the gene expression matrix from the top-ranked Final OS active cell population in the scRNA-seq dataset, converted it to a sparse matrix format, and constructed the phenoData and featureData based on cell metadata and gene information, respectively. These components were integrated into a Monocle object.For data normalization and differential analysis, we calculated size factors for each cell and estimated gene expression dispersion. Genes with high dispersion (defined as mean_expression ≥ 0.1 and dispersion_empirical ≥ dispersion_fit) were selected as highly variable genes, which served as feature genes for dimensionality reduction and trajectory inference.

Dimensionality reduction was performed using the DDRTree algorithm, and the results were used to construct pseudotime trajectories and cell ordering. Based on the pseudotime analysis, we further visualized the temporal expression dynamics of Final OS genes using scatter plots.

### Cell-cell communication analysis

CellChat is an R package designed to analyze intercellular communication based on scRNA-seq data annotated with distinct cell clusters. It leverages a curated database of ligand-receptor interactions for both human and mouse. In this study, we utilized CellChat along with the CellChatDB.human database to evaluate the major incoming and outgoing signaling patterns among different cell types in various sample groups.To visualize the global communication landscape, we employed the netVisual_circle function, which presents an overview of total cell-cell interactions and illustrates the strength and number of ligand-receptor pairs between the target cell cluster and other cell populations under different conditions.

### Statistical analysis

All statistical analyses were performed using R software (version 4.3.1). The Wilcoxon rank-sum test was used to compare differences between two groups. A p-value < 0.05 was considered statistically significant. Statistical significance levels are denoted as follows:

* P < 0.05, ** P < 0.01, *** P < 0.001, **** P < 0.0001.

## Results

### Elevated oxidative stress activity in IPF patients

**Figure 2:**
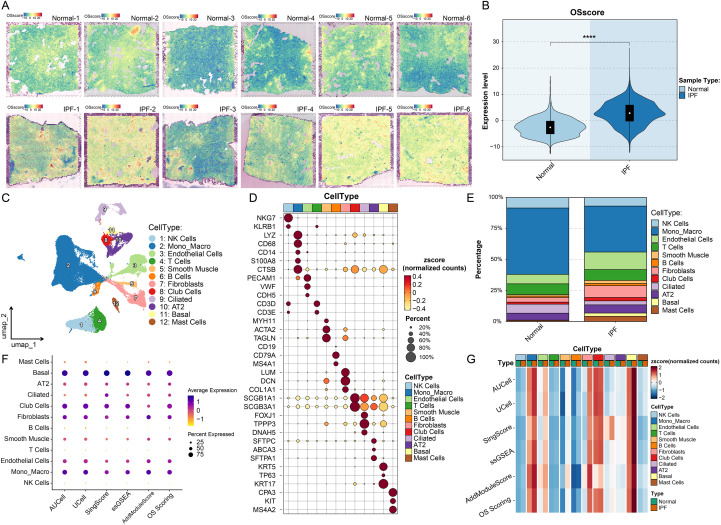
Spatial and single-cell landscape of oxidative stress activity in IPF lung tissue. **(A)** Spatial transcriptomic data were used to calculate oxidative stress (OS) scores for each spot across lung tissue sections from 6 healthy donors (Normal 1-6) and 6 IPF patients (IPF 1-6). OS activity was markedly elevated in IPF samples. **(B)** Violin plot comparing OS scores between normal and IPF groups across all spots, showing significantly higher OS levels in IPF (P < 0.0001). **(C)** Single-cell RNA-seq data (GSE128033) integrating 8 IPF and 10 normal lung samples (total of 73, 888 cells) were annotated into 12 major cell types. **(D)** Dot plot of representative marker genes for each cell type; dot color and size represent scaled expression level (z-score) and percentage of expressing cells, respectively. **(E)** Stacked bar plot showing the relative proportions of 12 cell types in normal vs. IPF groups, indicating no major differences in overall cell composition. **(F)** Bubble plot illustrating OS score distribution across cell types, with basal cells exhibiting the highest OS activity.G. Heatmap displaying OS scores in cells from normal and IPF groups across cell types. Most cell types in IPF samples showed increased OS activity, particularly basal cells, suggesting their potential role in oxidative stress-related IPF pathogenesis.

In the spatial transcriptomic data, we calculated oxidative stress (OS) scores for each spot in lung tissue sections derived from six healthy individuals and six patients with idiopathic pulmonary fibrosis (IPF). The results revealed that overall OS activity was markedly higher in IPF samples compared to normal tissues (**Figure 2A**). Further differential analysis between the two groups confirmed a significant elevation in OS activity in IPF lung regions (**Figure 2B**), suggesting a prominent oxidative stress status within fibrotic lesions that may contribute to disease progression.

Next, we conducted an in-depth analysis of oxidative stress-related features using the single-cell transcriptomic dataset GSE128033. Following standard quality control procedures, low-quality cells were excluded (**Supplementary Figures S1A, B**). Initial UMAP visualization revealed strong batch effects (**Supplementary Figure S1C**), which were effectively corrected using the Harmony algorithm. Ultimately, we integrated scRNA-seq data from 8 IPF patients and 10 healthy donors, yielding a total of 73, 888 high-quality single cells (**Supplementary Figure S1D**). Based on the top 3000 highly variable genes, dimensionality reduction and clustering identified 31 distinct cellular clusters (**Supplementary Figure S1E**). Using canonical marker genes (**Supplementary Figure S1F**), we manually annotated 12 major cell types, including NK cells, monocyte-macrophages, endothelial cells, T cells, smooth muscle cells, B cells, fibroblasts, Club cells, ciliated cells, alveolar type II (AT2) cells, basal cells, and mast cells (**Figure 2C**). Expression patterns of key marker genes are shown in **Figure 2D**, and the overall cell type composition was comparable between IPF and control groups (**Figure 2E**).

Upon completion of cell type annotation, we applied oxidative stress gene set-based enrichment scoring algorithms to quantify the OS activity of each cell. The distribution of OS scores across cell types is visualized in **Figure 2F**, showing that basal cells exhibited relatively high OS activity. To further explore differences in OS states between IPF and healthy samples, we plotted heatmaps comparing OS scores of cells from each cell type across the two groups (**Figure 2G**). The analysis demonstrated that OS scores were consistently higher in IPF-derived cells across all cell types, with basal cells displaying the most prominent elevation. This finding suggests that basal cells may undergo more severe oxidative stress responses in IPF and may play a central role in ROS-driven pathological remodeling.

### Cellular distribution of oxidative stress and candidate biomarkers identification in IPF

**Figure 3:**
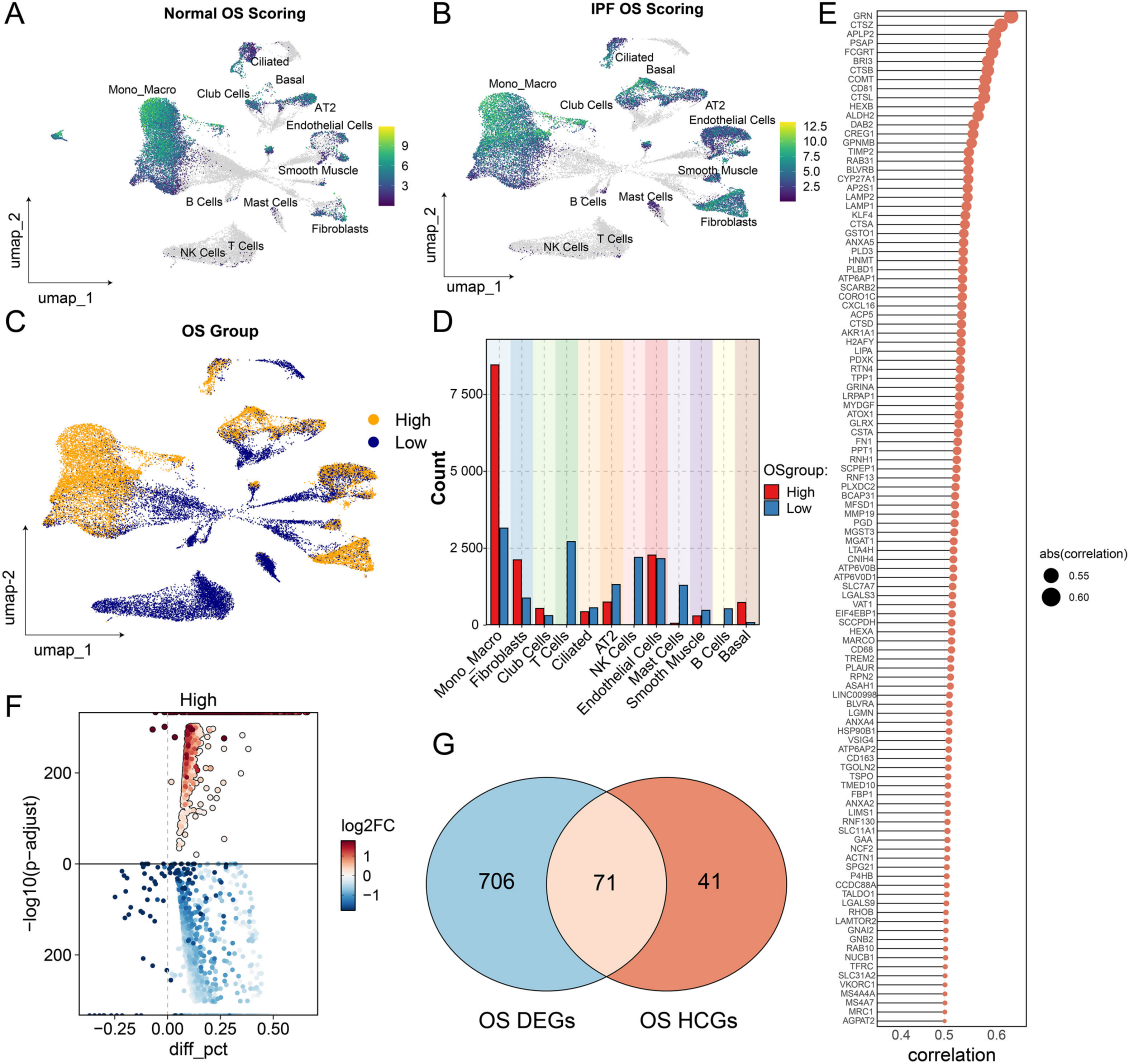
Distribution of high OS-active cells and identification of key factors in IPF lung tissue. **(A)** UMAP plot showing the distribution of oxidative stress (OS) scores in cells from normal lung tissue, where most cells exhibit low OS activity. **(B)** UMAP plot displaying OS score distribution in cells from IPF lung tissue, with elevated OS activity observed across multiple cell types. **(C)** IPF cells were divided into high OS activity group (OS high group) and low OS activity group (OS low group) based on the median OS score. UMAP plot visualizes their distribution within the cellular landscape. **(D)** Bar plot showing the number of OS high and OS low cells across cell types. Monocyte-macrophages had the highest number of OS high cells, whereas nearly all basal cells belonged to the OS high group. **(E)** Lollipop plot of representative genes positively correlated with OS scores; 112 OS high correlated genes (OS HCGs) were identified with correlation coefficient > 0.5. **(F)** Volcano plot of differentially expressed genes (OS DEGs) between OS high and OS low groups, revealing 777 OS DEGs. **(G)** Venn diagram showing the overlap between highly OS-correlated genes and OS-related DEGs, identifying 71 candidate key oxidative stress-associated genes.

After confirming the significant upregulation of oxidative stress (OS) activity in IPF lung tissues, we next explored the distribution of OS-high cells across different cell types and sought to identify key regulatory genes at the single-cell level. First, we visualized the distribution of OS scores separately in cells from healthy controls and IPF patients (**Figures 3A, B**), confirming that cells derived from IPF lungs exhibited a globally elevated OS status.Using the median OS score as a cutoff, we classified all single cells from IPF samples into two groups: OS-high and OS-low (**Figure 3C**). We then quantified the proportion of OS-high and OS-low cells within each annotated cell type (**Figure 3D**). Notably, monocyte-macrophages contained the largest number of OS-high cells, whereas nearly all basal cells belonged to the OS-high group, suggesting that this subpopulation may serve as a core responder under oxidative stress conditions.

To identify genes potentially involved in OS regulation, we first performed correlation analysis between gene expression and OS scores across all cells. A total of 112 genes showed strong positive correlations with OS scores (Pearson r > 0.5, p < 0.05) (**Figure 3E**), indicating that these may function as OS-associated regulators. Next, we conducted differential gene expression analysis between OS-high and OS-low cells, identifying 777 OS-related differentially expressed genes (OS-DEGs), including 610 genes significantly upregulated and 167 downregulated in OS^high cells (|logFC| > 1, p < 0.05) (**Figure 3F**; **Supplementary Table S2**). Finally, we intersected the 112 highly correlated genes with the 777 OS-DEGs and identified 71 core OS-associated candidate genes (**Figure 3G**). These genes were both highly associated with oxidative stress levels and differentially expressed under high OS activity, making them strong candidates for further biomarker discovery and mechanistic investigation in IPF pathogenesis.

### Robust diagnostic biomarker discovery for IPF via machine learning

**Figure 4:**
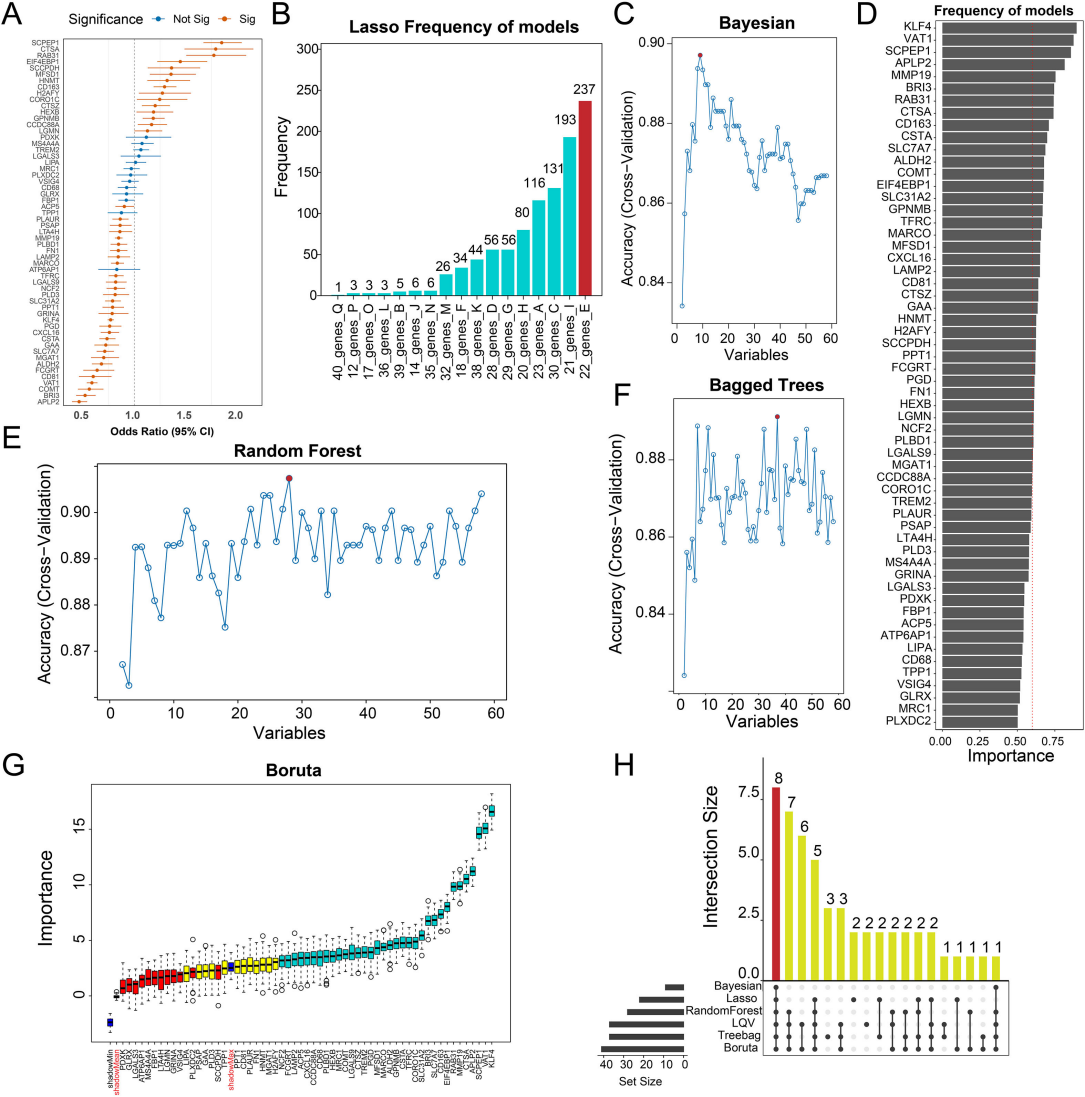
Identification of core OS-related features closely associated with IPF using multiple machine learning algorithms. **(A)** Univariate logistic regression analysis of 71 candidate oxidative stress (OS)-related genes, evaluating their association with IPF risk. Results are presented as odds ratios with 95% confidence intervals; orange bars indicate statistically significant variables. **(B)** LASSO regression model showing the frequency of gene selection across 1000 iterations with cross-validation; 22 high-frequency genes were retained. **(C)** Cross-validation accuracy curve for the Bayesian model under different variable counts; the optimal model included 9 variables. **(D)** Learning Vector Quantization (LVQ) model identifying 37 key genes based on their frequency of occurrence across multiple training iterations. **(E)** Random forest model performance across varying numbers of variables; the optimal model consisted of 28 genes. **(F)** Accuracy curve of the Bagged Trees model, with the best performance observed when 37 variables were included. **(G)** Boruta algorithm estimating variable importance; 41 important features were selected, with red-labeled genes representing the final intersecting features. **(H)** Upset plot showing intersections of selected genes from all 6 machine learning algorithms. 8 genes (APLP2, CD163, CTSA, KLF4, MMP19, RAB31, SCPEP1, VAT1) were consistently retained across all models and defined as the final OS-related signature genes (Final OS).

To identify key molecular factors closely associated with IPF pathogenesis, we used the bulk RNA-seq dataset GSE47460 as a training set and performed univariate logistic regression analysis based on disease status (IPF vs. healthy control). Among the 71 candidate biomarkers, 45 showed significant associations with IPF diagnosis (p < 0.05) (**Figure 4A**).To further narrow down robust diagnostic ones, we employed six classical machine learning algorithms: Least Absolute Shrinkage and Selection Operator (LASSO) regression, Bayesian modeling, Learning Vector Quantization (LVQ), Random Forest (RF), Bagged Trees (Treebag), and Boruta feature selection. These complementary approaches offer different advantages for variable selection and model stability.

In the LASSO regression analysis, 22 high-frequency genes were retained across 1000 iterations of cross-validation (**Figure 4B**). The Bayesian model achieved optimal predictive performance with 9 key variables (**Figure 4C**). LVQ selected 37 genes based on variable ranking (**Figure 4D**). The RF model identified 28 genes at the optimal accuracy threshold (**Figure 4E**), while the Treebag model selected 37 variables (**Figure 4F**). Boruta, which compares variable importance against shadow features, identified 41 significant features (**Figure 4G**).

By integrating results from all six models, we identified 8 overlapping genes:APLP2, CD163, CTSA, KLF4, MMP19, RAB31, SCPEP1, and VAT1 as the final diagnostic biomarkers (Final OS genes) (**Figure 4H**). These genes consistently showed high feature importance across different modeling frameworks, indicating their strong potential as stable and high-value biomarkers for IPF diagnosis.

### SCPEP1 serves as a robust IPF biomarker enriched in basal cells

**Figure 5:**
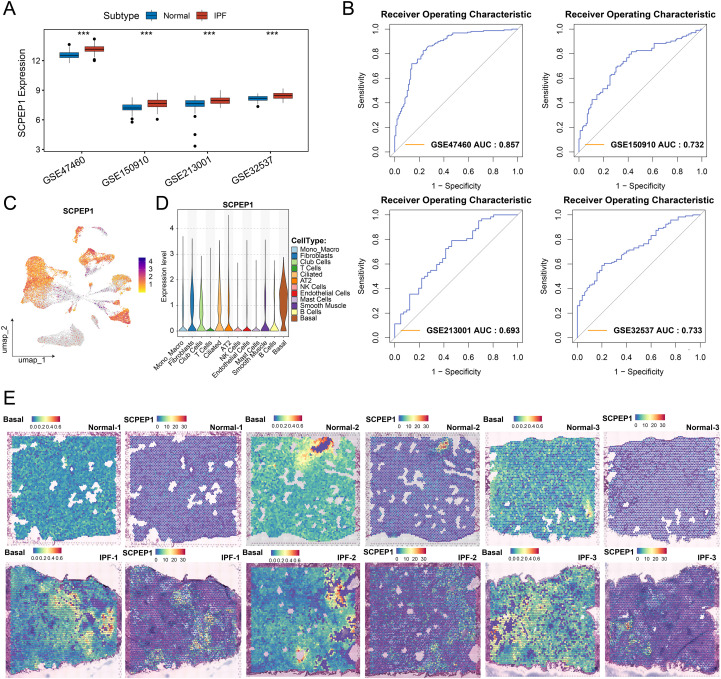
SCPEP1 Is a robust predictor of IPF events with cell-type and spatial specificity. **(A)** Boxplots showing the expression levels of SCPEP1 in the training dataset GSE47460 and validation datasets GSE150910, GSE213001, and GSE32537. SCPEP1 expression was significantly elevated in IPF samples compared to normal controls across all datasets. **(B)** Receiver operating characteristic (ROC) curves assessing the predictive power of SCPEP1 for IPF across four datasets, all demonstrating favorable discrimination performance. **(C)** Feature plot from single-cell transcriptomic data showing that SCPEP1 is preferentially expressed in basal cells. **(D)** Violin plots illustrating the distribution of SCPEP1 expression across different cell types, with basal cells exhibiting the highest expression. **(E)** Spatial transcriptomics maps showing that SCPEP1 expression is spatially co-localized with basal cell distribution. In normal tissues, SCPEP1 expression is relatively low, while in IPF tissues, its expression is markedly increased and predominantly enriched around airway regions, consistent with basal cell localization. Statistical comparisons were performed using the two-sided Wilcoxon rank-sum test. P < 0.05 was considered statistically significant (*P < 0.05, **P < 0.01, ***P < 0.001, ns P ≥ 0.05).

Among the 8 candidate biomarkers identified through machine learning, SCPEP1 emerged as the only gene consistently and significantly upregulated in IPF samples across the training dataset (GSE47460) and three independent validation cohorts (GSE150910, GSE213001, and GSE32537) (**Figure 5A**; **Supplementary Figures S2A, D**). This consistent elevation suggests that SCPEP1 may serve as a more stable marker of disease occurrence in IPF. To evaluate its diagnostic potential, we constructed receiver operating characteristic (ROC) curves using all four datasets. SCPEP1 demonstrated favorable predictive performance, with area under the curve (AUC) values of 0.857, 0.732, 0.693, and 0.733, respectively (**Figure 5B**), indicating its reliability as a diagnostic indicator.

To determine the cell type-specific expression of SCPEP1, we examined its distribution in single-cell RNA-seq data. Feature plots revealed that SCPEP1 expression was predominantly restricted to basal cells (**Figure 5C**). This finding was further validated by statistical comparison, which showed that SCPEP1 expression in basal cells was significantly higher than in any other cell type (**Figure 5D**). We further reconstructed the spatial distribution of basal cells using spatial transcriptomic data and SPOTlight deconvolution analysis. The results indicated that basal cells were primarily located around airway structures. Spatial co-localization analysis showed that SCPEP1 expression overlapped highly with basal cell-enriched regions in IPF tissues, and was lower expressed in normal tissues (**Figure 5E**; **Supplementary Figure S2E**).

**Figure 6:**
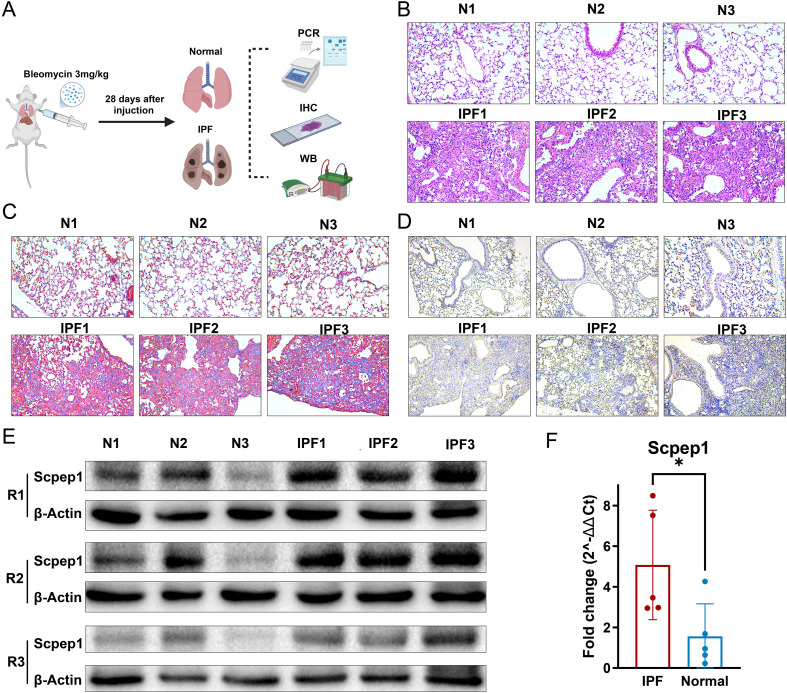
Validation of SCPEP1 upregulation in a bleomycin-induced pulmonary fibrosis mouse model. **(A)** Schematic of the experimental design: mice were administered bleomycin (3 mg/kg) via intratracheal instillation and sacrificed after 28 days for downstream analyses including qPCR, immunohistochemistry (IHC), and Western blot (WB). **(B)** Representative hematoxylin and eosin (H&E) staining images of lung tissues from normal (N1-N3) and bleomycin-induced IPF mice (IPF1-IPF3), showing alveolar collapse, interstitial thickening, and inflammatory cell infiltration in the IPF group. **(C)** Masson’s trichrome staining reveals extensive collagen deposition (blue) in the lungs of IPF mice, compared to minimal staining in normal lungs. **(D)** Immunohistochemical staining of SCPEP1 shows markedly increased expression in IPF lungs, particularly in airway epithelial and interstitial regions, compared to weak staining in normal controls. **(E)** Western blot analysis of SCPEP1 expression in lung tissues from normal and IPF mice across three biological replicates (R1-R3). β-actin was used as a loading control. **(F)** Quantitative real-time PCR analysis of Scpep1 mRNA expression. Data are presented as fold change calculated by the 2^–ΔΔCt^ method. Scpep1 was significantly upregulated in the IPF group compared to the normal group (* indicate P(0.0365) < 0.05, two-tailed unpaired t-test; t = 2.508, df = 8. The mean difference was −3.524 ± 1.405 (95% CI: −6.763 to −0.2842), with a large effect size (η² = 0.4402)).

For experimentally validating these findings, we established a bleomycin-induced pulmonary fibrosis model in C57BL/6 mice. Lung tissues were collected on day 28 following intratracheal administration and subjected to histological and molecular analyses (**Figure 6A**). Hematoxylin and eosin (H&E) staining revealed that, compared to the normal group (N1-N3), lung tissues from the IPF model group (IPF1-IPF3) exhibited marked architectural disruption, alveolar collapse, interstitial thickening, and increased inflammatory cell infiltration, all indicative of typical fibrotic pathology (**Figure 6B**). Masson’s trichrome staining was further performed to assess collagen deposition (**Figure 6C**). A substantial accumulation of collagen fibers (blue staining) was observed in the interstitial and perivascular regions of the IPF group, which was markedly greater than in the control group, confirming successful induction of pulmonary fibrosis by bleomycin.

To evaluate the expression pattern of SCPEP1 at the tissue level, immunohistochemical (IHC) staining was performed (**Figure 6D**). SCPEP1 expression was obviously elevated in the IPF group, with enhanced staining intensity and expanded distribution, particularly in the airway epithelium and interstitial compartments. In contrast, only weak expression was detected in the normal lung tissues. These findings further support the potential role of SCPEP1 in the pathogenesis of pulmonary fibrosis and suggest its involvement in pathological tissue remodeling potentially associated with oxidative stress. To further validate the expression of Scpep1 at both the protein and transcriptional levels, we performed and Western blotting (WB) quantitative real-time PCR (qPCR) using lung tissues from the bleomycin-induced pulmonary fibrosis model. Western blot analysis showed robust elevation of SCPEP1 protein expression in IPF samples relative to normal controls (**Figure 6E**; **Supplementary Figure S4**). This upregulation was reproducible across three biological replicates (R1-R3), with β-actin used as the internal loading control. Consistently, qPCR analysis revealed a significant upregulation of Scpep1 mRNA in the IPF group compared to the normal control group (**Figure 6F**). The fold change analysis (2^–ΔΔCt^) demonstrated that Scpep1 transcript levels were markedly increased in fibrotic lung tissues, supporting the transcriptomic prediction.

This indicates that SCPEP1 expression in IPF lungs is not only disease-specific but also exhibits strong spatial and cellular specificity, largely driven by basal cells.

### Potential role of SCPEP1^+^ basal cells in IPF-associated signaling and communication

**Figure 7:**
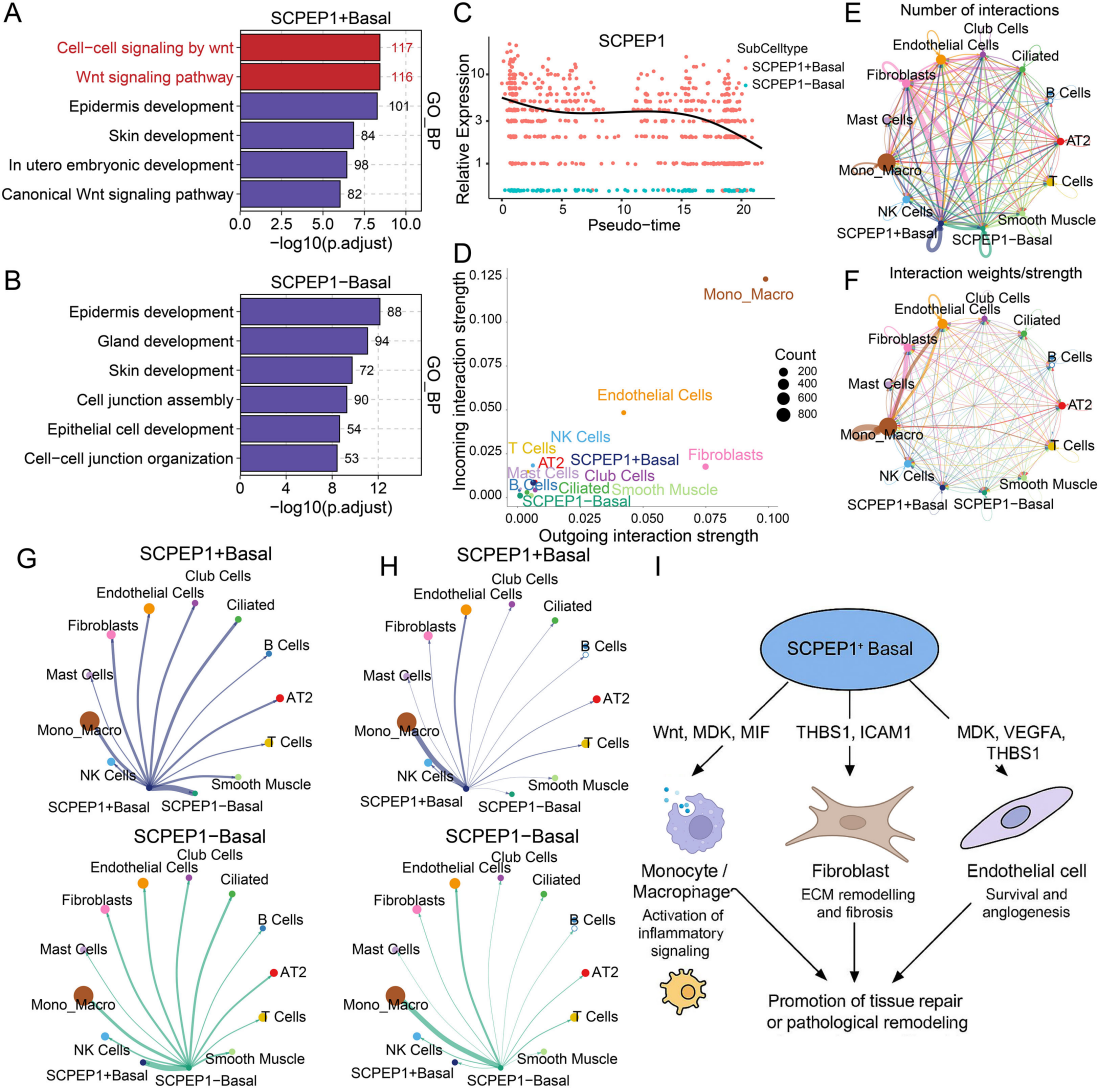
Multifaceted roles of SCPEP1^+^ basal cells in IPF progression. **(A)** Bar plot of GO Biological Process enrichment analysis reveals that SCPEP1^+^ Basal cells are significantly enriched in Wnt-related signaling pathways. **(B)** Enrichment analysis of SCPEP1^−^ Basal cells shows involvement in epithelial homeostasis-related processes. **(C)** Pseudotime scatter plot illustrates the dynamic expression of SCPEP1 during the differentiation trajectory of Basal cells, displaying an overall trend of initial upregulation followed by downregulation. **(D)** Distribution plot of incoming and outgoing signaling strengths among cell types shows that SCPEP1^+^ Basal cells possess stronger interaction capabilities compared to SCPEP1^−^ Basal cells. **(E)** Network diagram showing the number of distinct signaling pathways exchanged between cell types. **(F)** Network diagram displaying the overall interaction strengths between different cell types. **(G)** Shell plot visualizing the number of outgoing signaling pathways from Basal cells to other cell types; SCPEP1^+^ Basal cells are involved in a more complex communication network. **(H)** Shell plot visualizing the strength of outgoing signaling from Basal cells to other cell types. **(I)** Schematic diagram illustrating the proposed role of SCPEP1^+^ Basal cells in promoting tissue repair or pathological remodeling during IPF progression.

To systematically explore the potential mechanisms by which SCPEP1 functions within basal cells during IPF progression, we stratified basal cells into SCPEP1^+^ and SCPEP1^−^ subpopulations based on SCPEP1 expression. Gene Ontology biological process (GO_BP) enrichment analysis revealed that SCPEP1^+^ basal cells were significantly enriched in Wnt-related signaling pathways, including “cell-cell signaling by Wnt”, “Wnt signaling pathway” and “canonical Wnt signaling pathway” (**Figure 7A**). These cells were also associated with processes such as “epidermis development”, “skin development” and “*in utero* embryonic development, “ suggesting that SCPEP1^+^ basal cells may represent a developmentally reprogrammed and plastic population capable of mediating aberrant repair responses under chronic stress. In contrast, SCPEP1^−^ basal cells were enriched in pathways related to epithelial structural maintenance and homeostasis, including “cell junction assembly”, “epithelial cell development” and “cell–cell junction organization” (**Figure 7B**). These results imply that SCPEP1^−^ basal cells are more functionally aligned with barrier preservation, whereas SCPEP1^+^ basal cells may exhibit a more activated, remodeling-prone phenotype.

Pseudotime trajectory analysis further demonstrated that SCPEP1^+^ basal cells showed dynamic expression patterns along the developmental axis, with SCPEP1 expression gradually decreasing as pseudotime advanced (**Figure 7C**). This suggests that SCPEP1^+^ basal cells may be transiently activated early in the fibrotic response and subsequently transition to a quiescent or exhausted state.

To elucidate their role in intercellular communication, we constructed a global cell-cell interaction map. The overall communication strength (**Figure 7D**) indicated that SCPEP1^+^ basal cells exhibited higher incoming and outgoing signaling activity compared to SCPEP1^−^ basal cells. Network analysis revealed that fibroblasts engaged in the greatest diversity of signaling pathways, while monocyte-macrophages exhibited the highest interaction strength (**Figures 7E, F**). Focusing on basal cells, SCPEP1^+^ cells were involved in a broader range of ligand-receptor pairs with other cell types (**Figure 7G**), although the overall interaction strength was comparable to that of SCPEP1^−^ cells (**Figure 7H**), suggesting that SCPEP1^+^ basal cells may participate in more complex and diverse modes of communication. Detailed ligand-receptor analysis revealed that SCPEP1^+^ basal cells actively engaged in several IPF-relevant signaling axes, including pro-inflammatory MIF–CD74, pro-fibrotic and growth factor pathways MDK–NCL/SDC1, and adhesion/remodeling pathways such as THBS1–CD47/ITGB1 and ICAM1–ITGAL (**Supplementary Figure S3A, B**). Together with the GO enrichment results, these findings suggest that SCPEP1^+^ basal cells are capable of sensing oxidative stress cues and orchestrating microenvironmental remodeling through inflammation, matrix reorganization, and angiogenic signaling (**Figure 7I**).

In summary, SCPEP1^+^ basal cells exhibit a transcriptionally reprogrammed phenotype and function as active nodes within the fibrotic niche. By participating in multifaceted communication with immune and stromal cells, they may serve as candidate intermediaries linking oxidative stress responses to tissue remodeling in IPF.

## Discussion

In this study, we integrated multi-omics datasets—including single-cell RNA sequencing, spatial transcriptomics, and bulk RNA-seq to comprehensively profile oxidative stress (OS) activity in idiopathic pulmonary fibrosis (IPF) and identify SCPEP1 as a robust OS-related biomarker. Recent studies have identified SCPEP1 as a lysosomal serine carboxypeptidase (33) broadly expressed across multiple tissues, including lung and heart, and in models of vascular remodeling, supporting its broader role in tissue repair and remodeling (34). Proteomic evidence also indicates SCPEP1 is elevated during lysosomal or proteostasis stress, which often accompanies oxidative stress (33, 35).We further demonstrated that SCPEP1^+^ basal cells exhibit distinctive transcriptional, spatial and intercellular communication characteristics that may mediate IPF progression. These findings not only enhance our understanding of the ROS-cell interaction axis in fibrosis but also provide potential insights and candidate targets for ROS-based therapeutic interventions.

First, our multi-platform analysis revealed widespread activation of oxidative stress in IPF, consistently observed across spatial, bulk, and single-cell transcriptomic levels. This elevated OS activity was not only present at the tissue scale but also enriched within specific cell types, particularly basal cells. Basal cells, a progenitor-like epithelial population located near the airways, have recently attracted attention for their role in lung repair and fibrosis (36–39). While prior studies have primarily focused on fibroblasts or alveolar epithelial cells in response to ROS (16, 40), our findings suggest that basal cells are also highly responsive to oxidative stress and may serve as critical factors for ROS-driven pathological remodeling in IPF.

To identify reliable oxidative stress-associated biomarkers, we implemented a comprehensive machine learning strategy incorporating 6 complementary algorithms. This ensemble approach minimized model-specific bias and enhanced robustness. Among the 8 intersecting genes identified, SCPEP1 emerged as a top candidate, showing consistent upregulation across multiple independent datasets and excellent diagnostic performance (AUC up to 0.857). Its increased expression was validated at both the mRNA and protein levels in a bleomycin-induced pulmonary fibrosis C57BL/6 mouse model. These findings suggest that SCPEP1 is not merely a marker of altered expression, but may be involved in pathways relevant to the pathogenesis of IPF. While previous reports have linked SCPEP1 to vascular remodeling and inflammation (41), our study is the first to directly associate SCPEP1 with IPF and oxidative stress responses, thus expanding the known functional repertoire of this gene.

We also demonstrated that SCPEP1 expression is highly specific to basal cells and significantly elevated in IPF. Basal cells are recognized as facultative progenitors that repopulate the damaged airway epithelium, and under chronic stress, their dysregulated activation may lead to maladaptive remodeling (42–44). Notably, SCPEP1^+^ basal cells exhibited transcriptional signatures enriched in Wnt signaling, epithelial regeneration, and developmental reprogramming, suggesting a highly plastic and reactive state and may adopting a regenerative-like phenotype that becomes pathological in fibrotic contexts. Pseudotime trajectory analysis revealed that SCPEP1 is highly expressed during the early stages of cell fate progression and subsequently declines. This dynamic expression pattern aligns with its spatial localization near the airways in IPF tissues, suggesting that SCPEP1^+^ basal cells may represent early responders to injury, potentially initiating repair programs that become maladaptive under chronic stress.

Furthermore, intercellular communication analysis revealed that SCPEP1^+^ basal cells serve as active signal-sending and -receiving hubs within the fibrotic microenvironment. They engage in multiple IPF-relevant signaling pathways, including pro-inflammatory axes (MIF–CD74), pro-fibrotic/growth factor signals (MDK–NCL), and ECM remodeling interactions (THBS1–CD47, ICAM1–ITGAL). These results suggest that SCPEP1^+^ basal cells not only perceive ROS-associated environmental cues but also contribute to local inflammation, angiogenesis, and matrix reorganization, thereby acting as key intermediaries linking oxidative stress to fibrotic remodeling. Moreover, the spatial localization of SCPEP1^+^ basal cells near airway-adjacent vasculature raises the possibility of epithelial-vascular crosstalk. This interface is increasingly appreciated as a hypoxia- and oxidative stress-sensitive niche that facilitates pro-fibrotic signaling and endothelial cell activation (45, 46). SCPEP1^+^ basal cells may contribute to this process via paracrine signaling and matrix remodeling, potentially influencing endothelial-to-mesenchymal transition and amplifying fibroproliferative cascades. Although our current data are correlative, these observations position SCPEP1^+^ basal cells as plausible mediators of epithelial-vascular interaction in IPF, warranting further investigation. Collectively, SCPEP1^+^ basal cells are characterized by elevated oxidative stress response, developmental-like transcriptional states, complex communication networks, and spatial localization near fibrotic regions. These features converge to support their role as multifaceted regulators of tissue remodeling in IPF. In contrast to previously described epithelial subsets such as KRT5^+^KRT17^+^ aberrant basaloid cells involved in epithelial senescence and maladaptive remodeling, or KRT8^+^CLDN4^+^CDKN1A^+^ transitional cells representing arrested AT2-to-AT1 intermediates (37, 38, 42), SCPEP1^+^ basal cells not only act as ROS-sensing responders to tissue injury, but may also contribute to local fibrosis and immune dysregulation through active paracrine signaling. By integrating spatial transcriptomics and cell-level expression features, we propose for the first time that SCPEP1^+^ basal cells may serve as a candidate regulatory hub within the pathological remodeling of IPF. SCPEP1 shows potential as a clinically useful biomarker for IPF, given its cell-type specificity, spatial enrichment, and consistent upregulation. Its involvement in multiple pro-fibrotic signaling axes also highlights it as a promising biomarker candidate that merits evaluation for patient stratification and, in the longer term, for targeted intervention.

Our study offers several methodological advantages: the use of multi-omics integration ensured consistency across cellular, spatial, and tissue-level observations; multi-algorithmic biomarker discovery improved robustness and reproducibility; and spatial transcriptomics allows three-dimensional mapping of cells, gene expression, and anatomical context, offering direct evidence to connect molecular mechanisms with tissue-level pathology. Nevertheless, this study has some limitations. First, the functional role of SCPEP1 requires further validation through *in vitro* or *in vivo* perturbation experiments, as our current data are primarily correlative. Second, while our integrative multi-omics analyses reveal a strong association between SCPEP1 expression and IPF-related signaling pathways, we acknowledge that these findings are solely based on transcriptomic inference. Future studies employing perturbation approaches such as knockdown, overexpression, or spatial co-localization and ligand-receptor blocking assays will be essential to confirm causality and elucidate mechanistic underpinnings. Third, species differences between human and murine expression patterns must be carefully considered, as our wet-lab validation was performed in a mouse model of pulmonary fibrosis. Cross-species conservation of SCPEP1 function remains to be confirmed using human organoid systems or ex vivo models. Finally, although we validated findings in multiple public datasets but clinical utility such as SCPEP1-guided patient stratification, precision diagnosis and therapeutic intervention needs to be confirmed in large prospective patient cohorts.

In conclusion, we constructed a comprehensive oxidative stress activity landscape in IPF and identified SCPEP1 as a novel OS-responsive factor that is specifically upregulated in basal cells. Our findings suggest that SCPEP1^+^ basal cells are associated with early epithelial responses and multicellular interactions that contribute to fibrotic remodeling. However, the functional role of SCPEP1 requires experimental validation in future perturbation studies. These insights broaden our understanding of IPF pathogenesis and offer a theoretical foundation for future developing individualized, ROS-targeted therapeutic strategies.

## Conclusion

In summary, our study leveraged the integrative power of bulk RNA-seq, single-cell transcriptomics, and spatial transcriptomics to construct a multi-dimensional oxidative stress landscape in idiopathic pulmonary fibrosis (IPF). Through a combination of machine learning algorithms and multi-omics validation, we identified SCPEP1 as a robust oxidative stress-related biomarker with diagnostic potential. Notably, SCPEP1 was specifically enriched in basal cells and dynamically expressed during early epithelial differentiation, with spatial localization near airway-adjacent fibrotic niches.

SCPEP1^+^ basal cells exhibited transcriptional plasticity, strong oxidative stress responsiveness and active participation in intercellular communication, particularly via pro-fibrotic and pro-inflammatory signaling pathways. These findings suggest that SCPEP1^+^ basal cells may serve as early injury-responsive initiators and regulatory readout that link oxidative stress to pathological remodeling in IPF.

Importantly, spatial transcriptomics enabled us to achieve a three-dimensional alignment of cell identity, gene expression, and anatomical context, providing direct visual evidence that bridges molecular mechanisms with tissue-level pathology. Our work not only expands the functional understanding of SCPEP1 but also highlights the importance of spatially resolved, cell-type-specific analysis in uncovering potential targets for precision diagnostics and therapeutic strategies in fibrotic lung diseases, which warrants future validation through functional perturbation experiments. Their involvement in key ligand-receptor axes offers opportunities for targeted interventions aimed at disrupting pathological epithelial-stromal signaling in IPF.

## Data Availability

The original contributions presented in the study are included in the article/**Supplementary Material**. Further inquiries can be directed to the corresponding author.

## Supplementary material

Supplementary Figure 1 Preprocessing of scRNA-seq Data. **(A)**. Scatter plots showing the distribution of mitochondrial gene percentage, RNA counts, and erythrocyte gene percentage before quality control (QC). **(B)**. Scatter plots showing the same metrics after QC filtering. **(C)**. UMAP plot of cell distribution before batch effect correction using Harmony. **(D)**. UMAP plot after Harmony-based batch correction showing improved integration across samples. **(E)**. UMAP visualization of 31 unsupervised clusters identified from the dataset. **(F)**. Bubble plot showing the expression levels of canonical marker genes across identified clusters; dot size represents the percentage of cells expressing the gene, and color indicates average expression level (z-score).

Supplementary Figure 2 Expression Profiles of Final OS Genes Across Datasets. **(A)**. Violin plots showing the expression of the 8 Hub OS genes in the GSE47460 dataset. All genes exhibited significant intergroup differences; CD163, CTSA, RAB31, and SCPEP1 were notably upregulated in IPF samples. **(B)**. Violin plots of the 8 Hub OS genes in the GSE150910 dataset. Except for CD163 and RAB31, the remaining genes showed significant differences between groups, with CTSA, MMP19, and SCPEP1 markedly elevated in IPF. **(C)**. Violin plots of the 8 Hub OS genes in the GSE213001 dataset. Except for CD163 and CTSA, other genes such as KLF4, MMP19, RAB31, and SCPEP1 were significantly upregulated in IPF samples. **(D)**. Violin plots of 5 Hub OS genes in the GSE32537 dataset. CD163, KLF4, SCPEP1, and VAT1 showed distinct expression differences between groups, among which SCPEP1 was the only gene significantly elevated in IPF. **(E)**. Spatial transcriptomic heatmaps displaying SCPEP1 expression and its overlap with Basal cell distribution. SCPEP1 expression was lower in normal tissues but markedly increased in IPF lungs, predominantly localized around airway regions and colocalized with Basal cell-enriched zones. Statistical comparisons were performed using the two-sided Wilcoxon rank-sum test. P < 0.05 was considered statistically significant (*P < 0.05; **P < 0.01; ***P < 0.001; ns = not significant).

Supplementary Figure 3 Cell-Cell Communication Features of Basal Cell Subsets. **(A)**. Bubble plot showing detailed intercellular communication between SCPEP1^+^ Basal cells and other cell types. **(B)**. Bubble plot illustrating intercellular communication between SCPEP1^−^ Basal cells and other cell types. The size of the bubbles represents the number of significant ligand-receptor pairs, and the color indicates interaction strength.

## Glossary

IPF: Idiopathic Pulmonary Fibrosis
OS: Oxidative Stress
ROS: Reactive Oxygen Species
ECM: Extracellular Matrix
EMT: Epithelial-to-Mesenchymal Transition
FMT: Fibroblast-to-Myofibroblast Transition
scRNA-seq: Single-Cell RNA Sequencing
stRNA-seq/ST: Spatial Transcriptomics
WB: Western Blot
qPCR: Quantitative Polymerase Chain Reaction
H&E: Hematoxylin and Eosin
AUC: Area Under the Curve
DEG: Differentially Expressed Gene
OS DEG: Oxidative Stress-related Differentially Expressed Gene
GO_BP: Gene Ontology - Biological Process
UMAP: Uniform Manifold Approximation and Projection
PCA: Principal Component Analysis
LASSO: Least Absolute Shrinkage and Selection Operator
LVQ: Learning Vector Quantization
BLM: Bleomycin
IHC: Immunohistochemistry.
