# Supplementary figures and images for: SCPEP1+ basal cells are associated with the remodeling of oxidative stress signaling networks in idiopathic pulmonary fibrosis
Source: Front Immunol. 2025 Dec 8;16:1676086. doi: 10.3389/fimmu.2025.1676086 (PMC12719287; doi:10.3389/fimmu.2025.1676086)

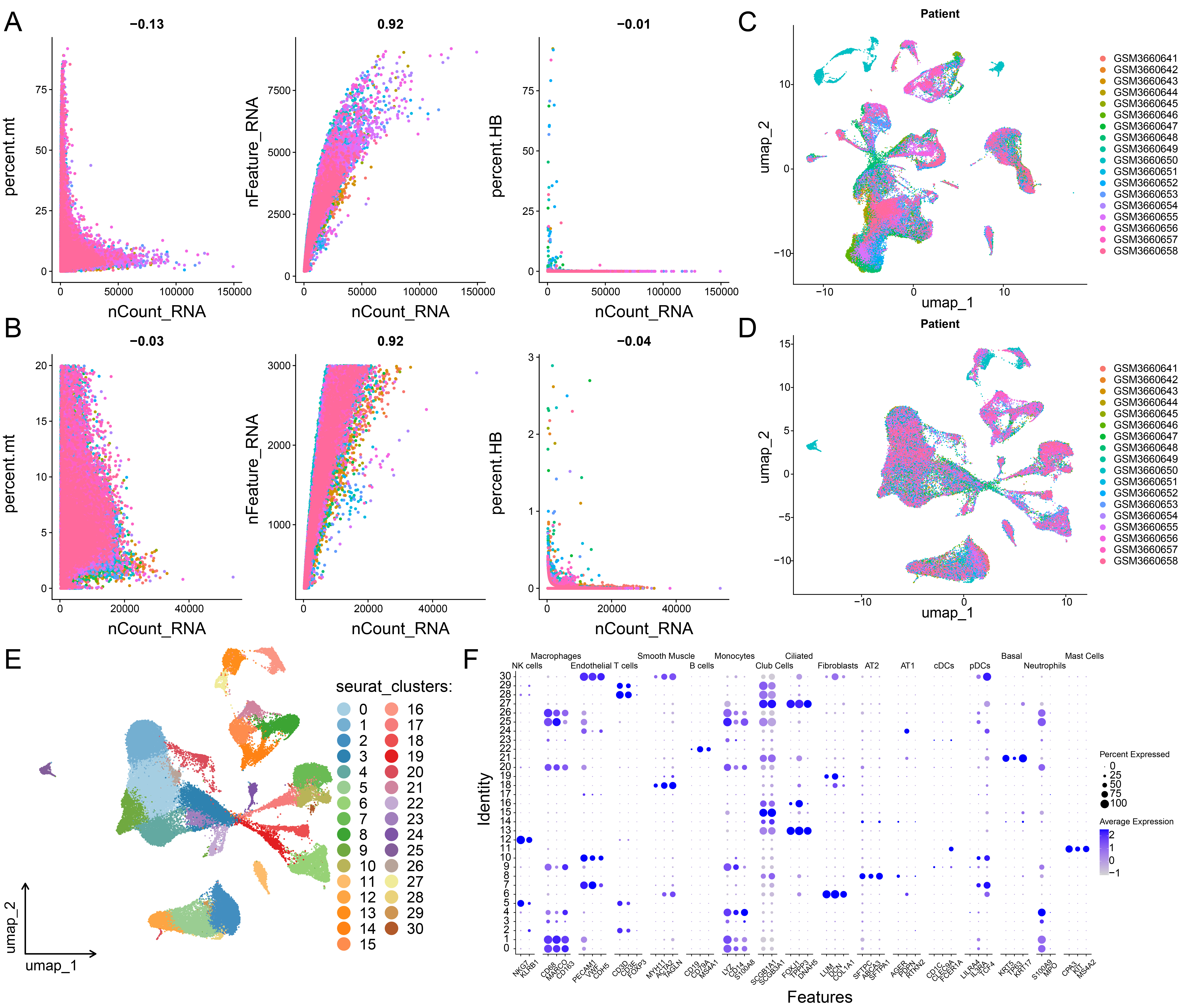

Supplement: Supplementary Figure 1 — Preprocessing of scRNA-seq Data. (A). Scatter plots showing the distribution of mitochondrial gene percentage, RNA counts, and erythrocyte gene percentage before quality control (QC). (B). Scatter plots showing the same metrics after QC filtering. (C). UMAP plot of cell distribution before batch effect correction using Harmony. (D). UMAP plot after Harmony-based batch correction showing improved integration across samples. (E). UMAP visualization of 31 unsupervised clusters identified from the dataset. (F). Bubble plot showing the expression levels of canonical marker genes across identified clusters; dot size represents the percentage of cells expressing the gene, and color indicates average expression level (z-score). [file Image1.jpeg]

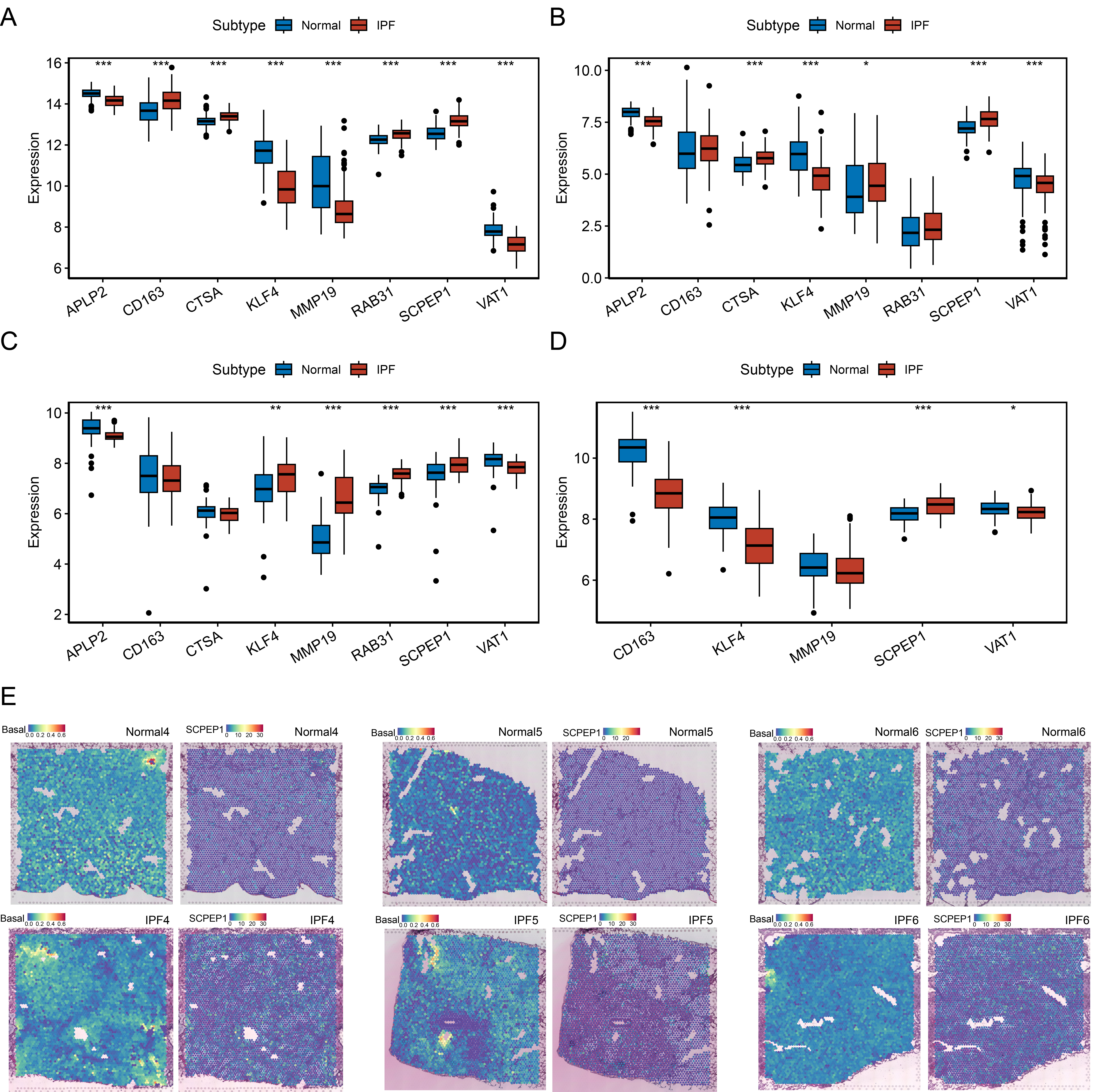

Supplement: Supplementary Figure 2 — Expression Profiles of Final OS Genes Across Datasets. (A). Violin plots showing the expression of the 8 Hub OS genes in the GSE47460 dataset. All genes exhibited significant intergroup differences; CD163, CTSA, RAB31, and SCPEP1 were notably upregulated in IPF samples. (B). Violin plots of the 8 Hub OS genes in the GSE150910 dataset. Except for CD163 and RAB31, the remaining genes showed significant differences between groups, with CTSA, MMP19, and SCPEP1 markedly elevated in IPF. (C). Violin plots of the 8 Hub OS genes in the GSE213001 dataset. Except for CD163 and CTSA, other genes such as KLF4, MMP19, RAB31, and SCPEP1 were significantly upregulated in IPF samples. (D). Violin plots of 5 Hub OS genes in the GSE32537 dataset. CD163, KLF4, SCPEP1, and VAT1 showed distinct expression differences between groups, among which SCPEP1 was the only gene significantly elevated in IPF. (E). Spatial transcriptomic heatmaps displaying SCPEP1 expression and its overlap with Basal cell distribution. SCPEP1 expression was lower in normal tissues but markedly increased in IPF lungs, predominantly localized around airway regions and colocalized with Basal cell-enriched zones. Statistical comparisons were performed using the two-sided Wilcoxon rank-sum test. P < 0.05 was considered statistically significant (*P < 0.05; **P < 0.01; ***P < 0.001; ns = not significant). [file Image2.jpeg]

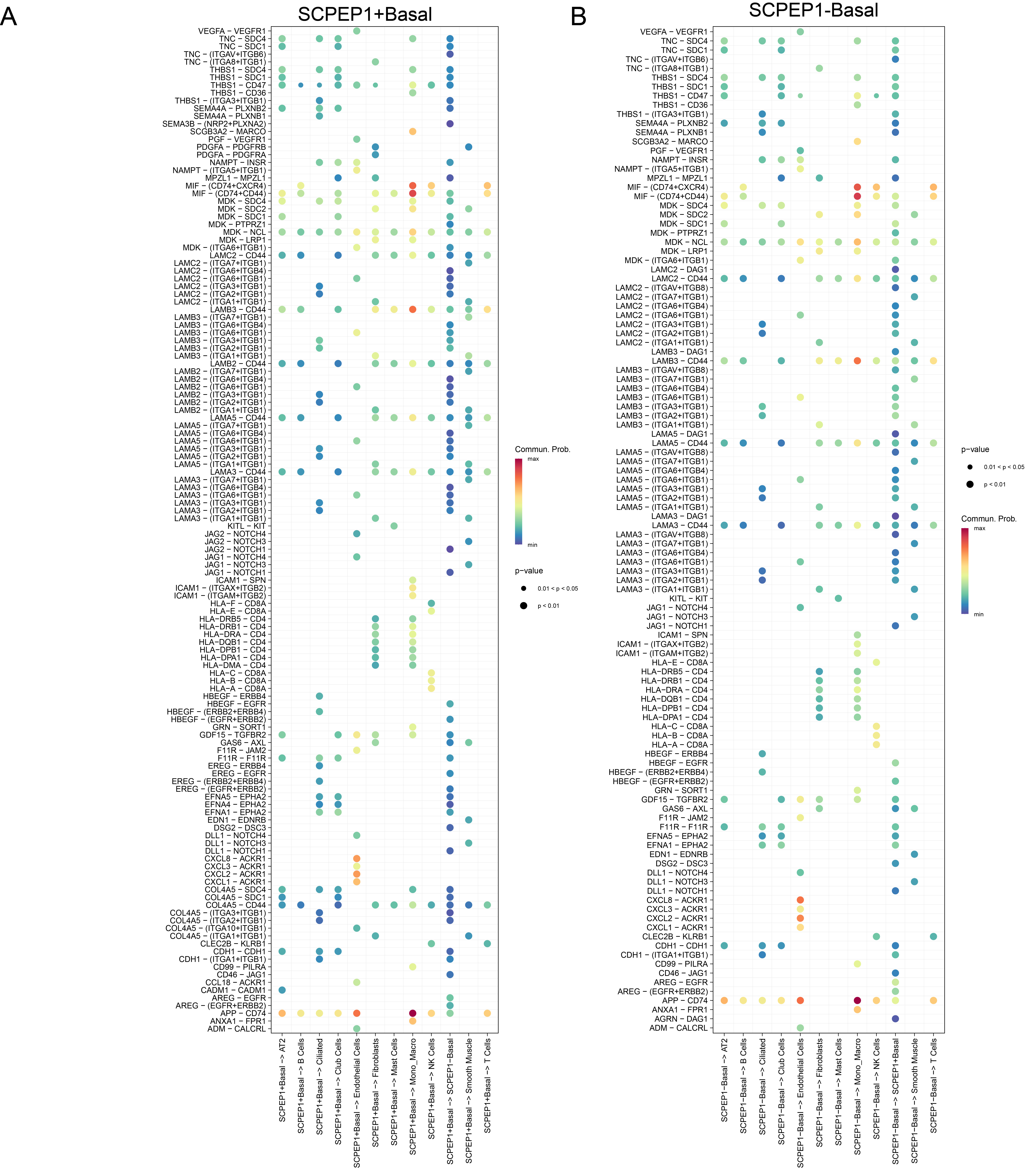

Supplement: Supplementary Figure 3 — Cell-Cell Communication Features of Basal Cell Subsets. (A). Bubble plot showing detailed intercellular communication between SCPEP1+ Basal cells and other cell types. (B). Bubble plot illustrating intercellular communication between SCPEP1− Basal cells and other cell types. The size of the bubbles represents the number of significant ligand-receptor pairs, and the color indicates interaction strength. [file Image3.jpeg]

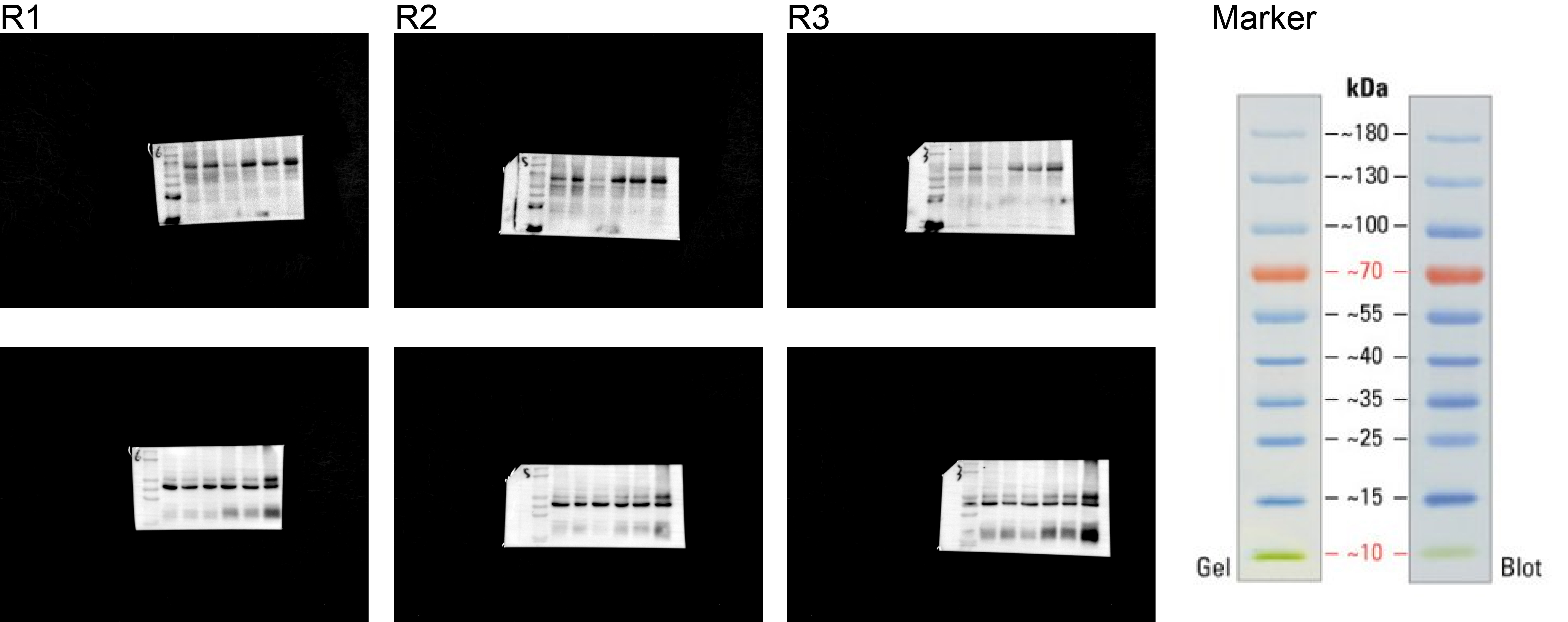

Supplement: Supplementary file 4 [file Image4.jpeg]
